# Limited impact of bacterial virulence on early mortality risk factors in *Acinetobacter baumannii* bacteremia observed in a *Galleria mellonella* model

**DOI:** 10.1038/s41598-024-65940-2

**Published:** 2024-06-28

**Authors:** Sin Young Ham, June Young Chun, Kyoung-Ho Song, Chang Kyung Kang, Jeong Su Park, Hee Bum Jo, Choong-Min Ryu, Yunsang Choi, Seong Jin Choi, Eunyoung Lee, Pyoeng Gyun Choe, Song Mi Moon, Wan Beom Park, Jihwan Bang, Sang-Won Park, Kyoung Un Park, Nam Joong Kim, Myoung-don Oh, Eu Suk Kim, Hong Bin Kim

**Affiliations:** 1 Division of Infectious Diseases, Department of Internal Medicine, Seoul National University Bundang Hospital, 82 Gumi-ro 173beon-gil, Bundang-gu, Seongnam-si, Gyeonggi-do 13620 Republic of Korea; 2 Department of Internal Medicine, National Cancer Center, Goyang, Republic of Korea; 3 Department of Internal Medicine, Seoul National University College of Medicine, Seoul, Republic of Korea; 4 Department of Laboratory Medicine, Seoul National University Bundang Hospital, Seongnam, Republic of Korea; 5 Division of Infectious Diseases, Department of Internal Medicine, Incheon Sejong Hospital, Incheon, Republic of Korea; 6 Infection Disease Research Center, KRIBB, Daejeon, Republic of Korea; 7 Department of Internal Medicine, Seoul Metropolitan Boramae Hospital, Seoul, Republic of Korea; 8 Present Address: Department of Internal Medicine, Korea Veterans Hospital, Seoul, Republic of Korea

**Keywords:** *Acinetobacter baumannii*, *Galleria mellonella*, Nosocomial infections, Early mortality, Bacteremia, Virulence, Infectious diseases, Bacteria, Pathogens

## Abstract

*Acinetobacter baumannii* (AB) has emerged as a major pathogen in vulnerable and severely ill patients. It remains unclear whether early mortality (EM) due to AB bacteremia is because of worse clinical characteristics of the infected patients or the virulence of the pathogen. In this study, we aimed to investigate the effect of AB virulence on EM due to bacteremia. This retrospective study included 138 patients with AB bacteremia (age: ≥ 18 years) who were admitted to a tertiary care teaching hospital in South Korea between 2015 and 2019. EM was defined as death occurring within 7 days of bacteremia onset. The AB clinical isolates obtained from the patients’ blood cultures were injected into 15 *Galleria mellonella* larvae each, which were incubated for 5 days. Clinical isolates were classified into high- and low-virulence groups based on the number of dead larvae. Patients’ clinical data were combined and subjected to multivariate Cox regression analyses to identify the risk factors for EM. In total, 48/138 (34.8%) patients died within 7 days of bacteremia onset. The Pitt bacteremia score was the only risk factor associated with EM. In conclusion, AB virulence had no independent effect on EM in patients with AB bacteremia.

## Introduction

*Acinetobacter baumannii* (AB) is a major causative pathogen of nosocomial infections^1^, particularly among critically ill patients and patients in the intensive care unit^2^. AB causes various hospital-acquired infections across several anatomical sites. Most commonly, AB infections manifest as ventilator-associated pneumonia or central line-associated bloodstream infections, and less frequently as skin and soft tissue infections, surgical site infections, and catheter-associated urinary tract infections^3^. The ability of AB to form biofilms enables it to move and attach to specific environments, and the breakdown of the host's anatomical barriers can lead to the development of bacteremia^4^. Once bacteremia develops, the host attempts to eradicate the bloodstream infection through an initial neutrophil response followed by additional actions from the complement system and macrophages. During this process, the lipopolysaccharide (LPS) of gram-negative bacteria, such as AB, is known to induce an excessive inflammatory response in the host, causing tissue and organ damage and rapidly worsening the patient's condition^5^. The virulence of AB and the host's immune status or underlying diseases^6^ are identified as reasons that make treating patients with AB bacteremia challenging. AB bacteremia accounts for approximately 17–62% of AB infections^7–9^. One study reported that early mortality (EM) due to bacteremia was as high as 61.5% of cases^10^. However, whether EM is attributable to the worse clinical characteristics of patients with AB bacteremia or to AB virulence remains unclear.

Bacterial virulence refers to the pathogen's capacity to cause disease by disrupting the host's normal physiological functions through various mechanisms. Traditionally, this virulence is categorized into four main groups of virulence factors, including adhesion and invasion, secretion systems, toxins, and iron acquisition. Efforts have been made to further classify these virulence factors into more detailed subcategories^11^. In AB, surface adhesins, glycoconjugates, secretion systems, and various other factors are known to directly influence pathogenesis, and corresponding virulence genes have been identified in previous studies^4^. However, experimental studies aimed at identifying virulence factors associated with EM in AB bacteremia are still lacking. Moreover, in clinical settings, various factors (including patient- and treatment-related factors) may all contribute to mortality; thus, specific genes or genomic sequence types alone are insufficient to explain mortality in AB cases.

The larval stage of the honeycomb moth, *Galleria mellonella*, has emerged as a useful insect model in research on host–pathogen interactions because of their low cost, ease of handling, survival at 37 °C, and similarities to mammals with respect to the innate immune system^12^. Several investigations on AB virulence have validated the *G. mellonella* model^13,14^. Therefore, this study aimed to investigate differences in the virulence among AB clinical isolates by inoculating *G. mellonella* larvae with AB obtained from patients with bacteremia and to identify the microbiological, demographic, and clinical factors affecting EM caused by AB bacteremia by combining AB virulence and patient clinical data.

## Results

### Patient characteristics

**Figure 1:**
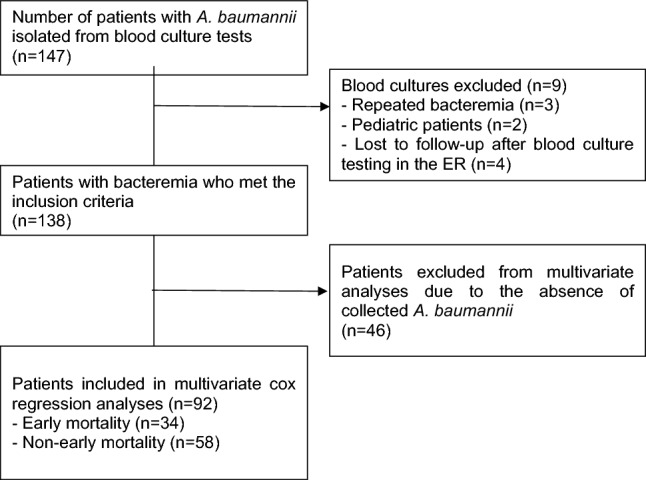
Flow diagram of patient selection. *A. baumannii*, *Acinetobacter baumannii*; *ER* emergency room.

Among the 147 patients who presented with AB bacteremia between 2015 and 2019, nine were excluded from the study; these included two children aged < 18 years, three patients with repeated bacteremia, and four patients who were transferred or discharged after blood culture testing in the emergency room. Finally, 138 patients were included in the study (Fig. 1). No epidemiologically relevant outbreaks of AB bacteremia occurred at the hospital during the study period.

**Figure 2:**
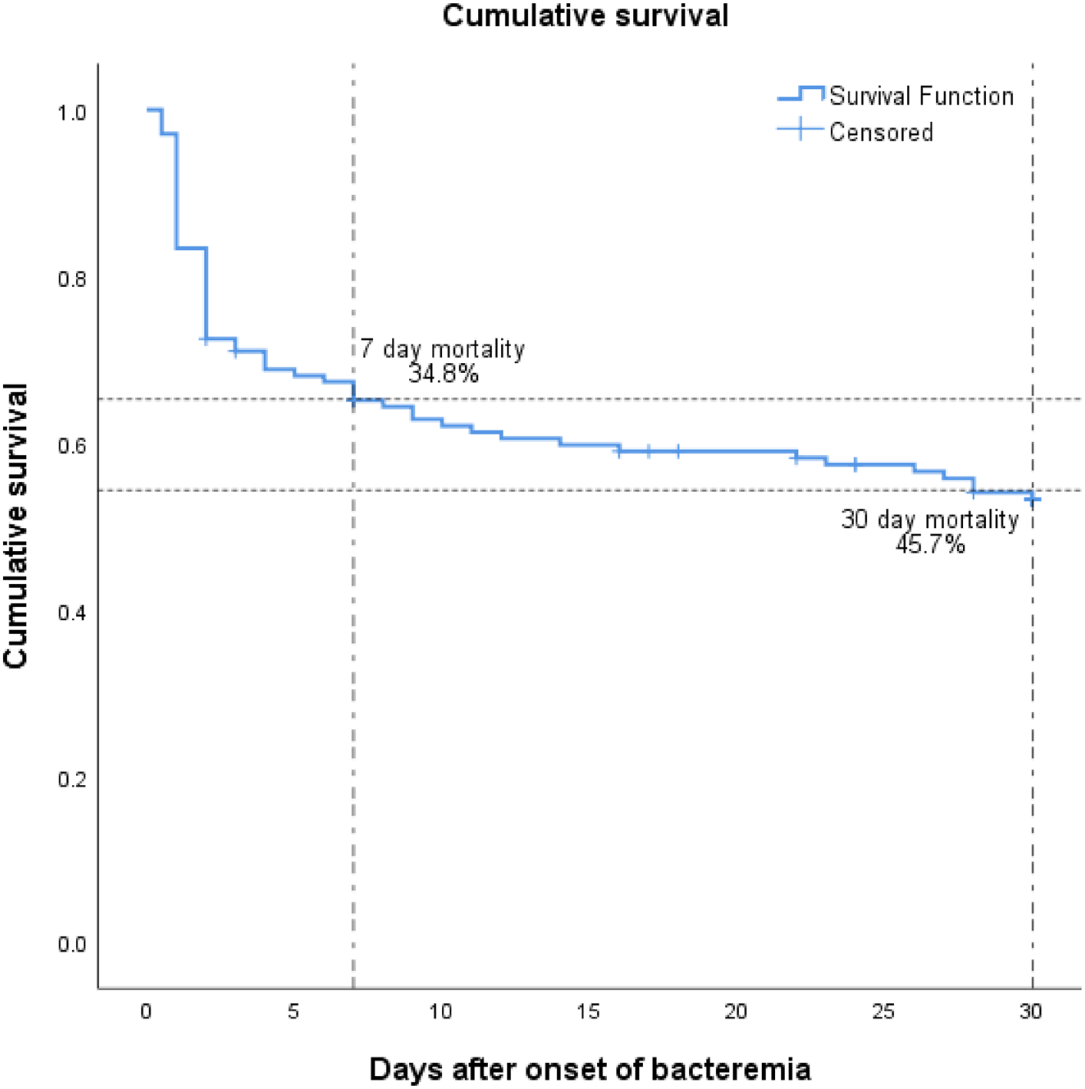
Thirty-day cumulative survival rate of patients with *Acinetobacter baumannii* bacteremia. The 7-day and 30-day mortality rates were 34.8% (48/138) and 45.7% (63/138), respectively.

Among these 138 patients, the 7-day and 30-day mortality rates were 34.8% (48/138) and 45.7% (63/138), respectively (Fig. 2). The 48 patients who died within 7 days comprised the EM group; the remaining 90 comprised the non-EM group.

AB was collected from 92 of the aforementioned 138 patients (clinical isolates-collected group). Data of all the variables collected did not differ significantly between the clinical isolates-collected and non-collected groups (Supplementary Table S1).

### Microbiological characteristics and virulence of AB

Of the 138 AB isolates with confirmed susceptibility, 79 (57.2%) were carbapenem-resistant AB (CRAB); the proportion of patients with CRAB was higher in the EM group than in the non-EM group (89.6 vs. 40.0%, *p* < 0.001). The antibiotic susceptibility data of the AB isolated from the blood cultures are presented in Supplementary Table S2. Ninety-two AB isolates were injected into *G. mellonella* larvae to obtain survival curves (Supplementary Figure S1). The K-means clustering analysis was based on the larval death count; accordingly, 92 AB isolates were divided into 2 virulence groups (Supplementary Table S3), namely the high virulence (HV; n = 50) and low virulence (LV; n = 42) groups.

### Risk factors for 7-day mortality in univariate and multivariate analyses

Univariate analyses were conducted on the demographic, clinical, treatment-related, and microbiological factors predicted to affect EM (Table 1). The mean age was higher in the EM group than in the non-EM group (71.81 vs. 66.92 years, *p* = 0.046). Conversely, the Charlson comorbidity-weighted index (CCI)^15^ did not differ significantly between the two groups (6.85 vs. 6.08, *p* = 0.131). Compared to in the non-EM group, more patients in the EM group had neutropenia (2.2 vs. 16.7%, *p* = 0.004) and were undergoing systemic steroid treatments (8.9 vs. 27.1%, *p* = 0.010). Furthermore, compared with the non-EM group, the EM group had a higher Pitt bacteremia score^16^ (2.70 vs. 7.06, *p* < 0.001) and Sequential Organ Failure Assessment (SOFA)^17^ score (5.20 vs. 12.94, *p* < 0.001) at bacteremia onset. The most common focus of infection in the EM group was lung infection (56.2%), followed by central line-associated infection (22.9%), and primary bacteremia (14.6%). Conversely, compared with the EM group, the non-EM group had a higher proportion of low-risk infection foci (*p* < 0.001): patients presented with central line-related infections (35.6%), primary bacteremia (20.0%), pancreaticobiliary infections (17.8%), and lung infections (12.2%). Compared to in the non-EM group, more patients in the EM group received inappropriate empirical antibiotics (53.3 vs. 83.3%,* p* = 0.001) and fewer patients underwent removal of the infection focus (24.4% vs. 4.2%, *p* = 0.006). In the early mortality group, the empiric antibiotics most frequently used were meropenem (31.0%), piperacillin/tazobactam (13.8%), and fluoroquinolones (10.3%). The empiric antibiotics used in both the early mortality and non-early mortality groups are listed in Supplementary Table S4. The variables predicted to be significant (*p* < 0.2) in Student’s *t*-test or chi-square test were analyzed using univariate and multivariate Cox regression analysis (Table 2).

**Table 1:** Clinical characteristics of patients with *Acinetobacter baumannii* bacteremia according to early mortality (n = 138).

	Non-early mortality (n = 90)		Early mortality (n = 48)		*p*-value
	n	(%)	n	(%)	
Age (y), mean ± SD	66.92 ± 14.19		71.81 ± 12.33		**0.046**
Male sex	55	(61.1)	36	(75.0)	0.147
Acquisition site					0.810
Community acquired	16	(17.8)	7	(14.6)	
Nosocomial^a^	74	(82.2)	41	(85.4)	
CCI, mean ± SD	6.08 ± 3.06		6.85 ± 2.44		0.131
Chronic lung disease	13	(14.4)	8	(16.7)	0.922
Rheumatologic disease	3	(339)	3	(6.2)	0.419
Leukemia	1	(1.1)	2	(4.2)	0.277
Lymphoma	0	(0)	3	(6.2)	**0.040**
Metastatic cancer	13	(14.4)	8	(16.7)	0. 922
Chronic kidney disease	19	(21.1)	18	(37.5)	0.062
Cerebrovascular disease	22	(24.7)	6	(12.8)	0.101
Chronic liver disease	19	(21.1)	6	(12.5)	0. 211
Diabetes mellitus	25	(27.8)	19	(40.4)	0.132
HIV infection	0	(0)	1	(2.1)	0.348
Neutropenia^b^	2	(2.2)	8	(16.7)	**0.004**
Recent chemotherapy	11	(12.2)	6	(12.5)	1.000
Systemic steroid	8	(8.9)	13	(27.1)	**0.010**
Other immunosuppressant	1	(1.1)	3	(6.2)	0.121
Pitt bacteremia score, mean ± SD	2.70 ± 2.74		7.06 ± 2.95		** < 0.001**
SOFA, mean ± SD	5.20 ± 4.05		12.94 ± 4.80		** < 0.001**
Infection focus					** < 0.001**
Primary bacteremia	18	(20.0)	7	(14.6)	
Lung	11	(12.2)	27	(56.2)	
Central-line associated	32	(35.6)	11	(22.9)	
Pancreaticobiliary	16	(17.8)	1	(2.1)	
Intra-abdominal	2	(2.2)	2	(4.2)	
Urinary tract	6	(6.7)	0	(0)	
Others	4	(4.4)	0	(0)	
Mortality risk of infection focus					**0.015**
Low risk^c^	41	(45.6)	11	(22.9)	
High risk^d^	49	(54.4)	37	(77.1)	
Inappropriate empirical antibiotics	48	(53.3)	40	(83.3)	**0.001**
Infection focus removal	22	(24.4)	2	(4.2)	**0.006**
Carbapenem resistance	36	(40.0)	43	(89.6)	** < 0.001**
Time to blood culture clearance (d), mean ± SD	1.48 ± 1.21		1.21 ± 0.58		0.080

*SD* standard deviation; *CCI* Charlson comorbidity index; *HIV* human immunodeficiency virus; *SOFA* Sequential Organ Failure Assessment.

^a^Nosocomial: 48 h after hospital admission, 3 days after discharge, or 30 days after surgery.

^b^Neutropenia: Absolute neutrophil count < 500/μL.

^c^Low-risk infection focus: infection focus with ≤ 30% associated mortality, including the urinary tract, intravenous catheter, and pancreaticobiliary tract.

^d^High-risk infection focus: infection focus with > 30% associated mortality, including the lungs, peritoneum, and unknown sources.

Significant values are in [bold].

**Table 2:** Results of the univariate and multivariate analyses for risk factors of early mortality in patients with *Acinetobacter baumannii* bacteremia (n = 92).

	Univariate analysis		Multivariate analysis	
	HR (95% CI)	*p*-value	Adjusted HR (95% CI)	*p*-value
Age	1.022 (0.993–1.051)	0.136	1.021 (0.988–1.056)	0.221
Sex	0.680 (0.317–1.458)	0.322		
CCI	1.066 (0.968–1.188)	0.247		
Immunosuppressed status^a^	1.909 (0.994–3.860)	0.072	1.376 (0.629–3.021)	0.425
Pitt score	1.343 (1.205–1.496)	** < 0.001**	1.278 (1.146–1.425)	** < 0.001**
High-risk infection focus^b^	2.849 (1.249–6.499)	**0.013**	2.450 (0.867–6.921)	0.091
Inappropriate empirical antibiotics	2.929 (0.211–7.088)	**0.017**	2.555 (0.996–6.554)	0.091
Infection focus removal	9.214 (1.259–67.435)	**0.029**	4.604 (0.555–38.183)	0.157
Carbapenem resistance	5.855 (2.058–16.658)	**0.001**	2.397 (0.806–7.126)	0.116
High virulence	1.864 (0.908–3.826)	0.090	1.311 (0.598–2.875)	0.499

*HR* hazard ratio; *CI* confidence interval; *CCI* Charlson comorbidity index; *LV* low virulence; *HV* high virulence.

^a^Immunosuppressed status: absolute neutrophil count < 500/μL, recent chemotherapy, use of steroids or immunomodulators.

^b^High-risk infection focus: infection focus with > 30% associated mortality, including the lungs, peritoneum, and unknown sources.

Significant values are in [bold].

Multivariate analyses were conducted on the 92 cases of bacteremia with virulence data obtained from a *G. mellonella* infection model. We selected variables deemed clinically significant following the univariate Cox regression analysis of the EM group. The adjusted hazard ratios (aHRs) were calculated using a Cox regression model (Table 2). In multivariate analysis, only the Pitt bacteremia score at bacteremia onset was related to EM (aHR = 1.278 at 1-point increase, 95% confidence interval [CI]: 1.146–1.425).

Mortality risk of infection foci, inappropriate empiric antibiotics, unremoved infection foci, and carbapenem resistance were significantly associated with EM in univariate analysis (*p* < 0.05) but not in multivariate analysis. The high virulence group tended to have an increased risk of EM in univariate analysis (HR = 1.864, 95% CI: 0.908–3.826), but there was no significant association in multivariate analysis (aHR = 1.311, 95% CI: 0.598–3.826).

## Discussion

A meta-analysis revealed that the 30-day mortality rates in AB bacteremia were 39.5%–83.7%^6^, with most studies considering 30-day mortality as the outcome. However, Lee et al.^10^ reported that 61.5% of the deaths within 30 days occurred within the first 3 days; in our study, 34.8% (48/138) of the patients died within 7 days. This indicates that AB bacteremia-related mortality peaks early after the onset of bacteremia. We focused on EM to investigate the effects of microbial factors, because patient clinical factors (rather than AB virulence) may exert a greater effect on late mortality in AB bacteremia. The outcome of interest was the 7-day mortality, reflecting the cumulative survival trend (Fig. 2) and the effects of potential microbiological and treatment-related factors on EM in AB bacteremia. According to Pena et al.^18^ the *Pseudomonas aeruginosa exoU* genotype is an independent factor for 5-day mortality in *P. aeruginosa* bacteremia. While specific genes associated with AB bacteremia-related mortality have not yet been identified, we sought to identify EM-related factors by using a *G. mellonella* model to classify the virulence group and then matching virulence data with clinical data.

Multivariate analysis revealed that only the Pitt bacteremia scores were correlated with EM. According to Du et al.^6^, treatment-related factors and the Pitt bacteremia score were the main predictors of mortality in CRAB infections. Like the results of previous studies, this study also confirmed that the Pitt bacteremia score was related to EM. However, infection foci, inappropriate empiric antibiotics, unremoved infection foci, and carbapenem resistance, which were predicted to be associated with EM in univariate analysis (*p* < 0.05), did not prove to be related to EM in multivariate analysis. Patients who died within 3 days after bacteremia onset accounted for 30.4% (42/138), and even if appropriate antibiotics were administered to these patients, the antibiotic concentration may not have reached therapeutic levels before death. Therefore, the effect of appropriate empiric antibiotic administration may have been underestimated. Resistance to carbapenem has been correlated with increased mortality in most studies. In the present study, however, no correlation between carbapenem resistance and EM was found. Additional multivariate analysis of 30-day mortality in this study (Supplementary Table S5) showed that carbapenem resistance was associated with 30-day mortality, confirming that carbapenem resistance was associated with bacteremia-related mortality but had no effect on EM. According to Son et al.^19^, non-eradicated focus was associated with 30-day mortality in CRAB bacteremia. In this study, there was no significant association between EM and focus removal, which may be because the number of patients who underwent focus removal in our study was low at 17.4%. The reason for the fewer patients who had the infection focus removed compared to that in other studies was the high proportion of infection focus that was anatomically difficult to remove together with the severity of the disease.

Various in vivo and in vitro models have been applied to elucidate the virulence of AB. Animal models, such as *G. mellonella*, *C. elegans*, zebrafish, and mice, allow for the assessment of overall virulence in a living organism, reflecting the complex interactions within the host^4^. In contrast, in vitro models, such as complement sensitivity assays, provide specific insights into how well bacteria can resist the host’s complement system, one of the immune defenses^20^. However, while these animal and in vitro models can help identify virulence factors or strains^3^, they have the limitation of not fully representing the host–pathogen interactions observed in humans. The purpose of this study was to identify virulence factors associated with EM. Thus, we aimed to replicate EM in animal experiments using clinical isolates to identify strains that cause EM. Furthermore, we sought to identify the common phenotypes or genotypes shared by these strains. Therefore, we chose *G. mellonella* as a relatively easy-to-handle and cost-effective animal model.

The number of *G. mellonella* larval deaths after inoculation with AB isolates was used to classify the AB isolates into high and low virulence groups. In univariate analysis, the HV group tended to be associated with EM, but there was no significant association in multivariate analysis. Lee et al.^10^ also reported that clinical severity score was the only variable significantly associated with AB bacteremia-related 3-day mortality. These results suggest that the impact of virulence on EM may have been underestimated because factors related to the patient's clinical severity have a relatively large impact on EM. Follow-up studies including more bacteremia cases and clinical isolates are needed to analyze the virulence factors associated with EM. Furthermore, by conducting additional in vitro assays to analyze cytokine or chemokine responses that appear to influence early organ failure in bacteremia, we expect to further identify strains or genotypes associated with EM^21^.

This study has some limitations. First, the number of cases was small, and clinical isolates were not collected for all cases. Therefore, some variables may have been overestimated or underestimated in relation to EM during statistical analysis. Despite the fact that clinical isolates were not collected for all cases, all collected variables were not significantly different between the clinical isolates collected and non-collected groups. Second, considering the time and cost, virulence experiments were performed once per isolate. However, we attempted to maintain consistency across the results by using identical positive and negative controls, which were chosen from representative clinical isolates in the HV and LV groups for each experiment; we only accepted relevant data after confirming their consistency with data from the control group. Third, the clinical data were collected retrospectively; therefore, we may have missed some data. However, the study institution is equipped with an automatic consultation system involving an infectious disease physician^22^, which was initiated when bacteria were isolated from a blood culture. Most records were reviewed from the time of the blood culture test, potentially compensating for the limitations of a retrospective study design. Fourth, research institutions do not routinely report AB separately from the *Acinetobacter calcoaceticus-baumannii* (ACB) complex without additional testing. While some studies have concluded that there are differences in virulence among species^23^, Nithichanon et al. reported in their paper that AB and *A. nosocomialis* were clinically similar in terms of severity and mortality^24^. Additionally, Chen et al. claimed that there were no clinical differences among genospecies within the ACB complex^25^. Although it was not possible to further classify *Acinetobacter* species, considering the clinical similarity between species, it is unlikely to significantly impact the interpretation of the study results. Despite these limitations, this study has strengths in that it reproduced the virulence of clinical isolates in a *G. mellonella* model, the outcomes of which were analyzed in combination with clinical data from patients with bacteremia.

In conclusion, AB bacteremia-related mortality occurs in the early phase; higher Pitt bacteremia scores affect EM, but the AB virulence group from *G. mellonella* model is not associated with EM due to AB bacteremia. Further studies on microbial and genomic factors, such as specific genes, sequence types, or host–pathogen interactions associated with EM due to AB bacteremia are warranted.

## Methods

### Patients

This retrospective study was conducted at the Seoul National University Bundang Hospital, a tertiary care teaching hospital in South Korea. We included patients with AB bacteremia (age: ≥ 18 years) who were admitted to the hospital between 2015 and 2019. Relevant literature was referenced to identify variables related to AB bacteremia-associated mortality^6,19,26–28^. Medical records were reviewed for data on the patients’ demographic and clinical characteristics, laboratory test results, microbiological findings, treatments, and treatment outcomes.

### Definitions

One or more AB isolated from a blood culture system were considered causative microorganisms of bacteremia, and only the first bacteremia episode was included in this study. Hospital-acquired infections were defined as those that occurred at 48 h after hospital admission, within 3 days after hospital discharge, or within 30 days after surgery^29^. The severity of underlying diseases was expressed using the CCI^15^. Immunosuppressant use was defined as receipt of any type of chemotherapy within 30 days, use of corticosteroids equivalent to ≥ 20 mg of prednisolone for > 1 week, or use of other immunosuppressants. The severity of the illness was assessed using the Pitt bacteremia and SOFA scores^16,17^. Primary foci of infection were classified into low- and high-risk foci^30^. CRAB was identified based on in vitro resistance to imipenem or meropenem^31^. Appropriate antibiotic treatment was defined as the treatment of bacteremia appropriately with antibiotics showing in vitro activity during susceptibility testing. Bacteremia onset was defined as the first positive blood culture; EM was defined as death occurring within 7 days of bacteremia onset.

### Identification and antimicrobial susceptibility testing

All blood culture samples were prepared in the hospital’s department of laboratory medicine using standard blood culture systems, namely the BACT/ALERT VIRTUO® system (bioMérieux, Marcy-l'Étoile, France) and BD BACTEC™ FX (Becton Dickinson, East Rutherford, NJ, USA). The VITEK 2 system (bioMérieux) was used for susceptibility testing, and data on the susceptibility of the isolates were recorded. Isolates with intermediate susceptibility were considered resistant.

### G. mellonella infection model

This model was established with reference to similar studies^32^. The clinical isolates obtained from the blood culture system were stored at − 70 °C until larval inoculation. The stored isolates (1 × 10^8^ CFU/mL) were injected into the rear left proleg of the *G. mellonella* larvae (Korea Research Institute of Bioscience and Biotechnology, Daejeon, the Republic of Korea), weighing 200–250 mg. Each isolate was inoculated into 15 larvae, which were incubated for 5 days at 37 °C. Overall, 92 AB isolates were tested over 23 trials. The following four controls were established: one larva inoculated with a reference AB (ATCC 19,606), one larva injected with sterile phosphate-buffered saline, one larva inoculated with a clinical isolate with the highest virulence, and one larva inoculated with a clinical isolate with the lowest virulence. The larvae were evaluated daily for 5 days after inoculation, and death was defined as no response to touch.

### Statistical analyses

Continuous variables were analyzed using the Student’s *t*-test, whereas nominal variables were analyzed using the chi-square test or the Fisher exact test. The variables that affected EM were tested using a Cox regression analysis. A K-means clustering analysis was performed to classify the injected AB clinical isolates into virulence groups. *p* < 0.05 was considered significant. All statistical analyses were performed using IBM SPSS Statistics for Windows, version 26 (IBM Corp., Armonk, NY, USA).

### Ethics statements

This study received approval from the Institutional Review Board (IRB) of Seoul National University Bundang Hospital (SNUBH) (IRB number: B-1909-565-305). All research procedures were conducted in accordance with relevant guidelines and regulations, including the Declaration of Helsinki. Informed consent was waived by the IRB of SNUBH due to the retrospective nature of the study.

### Supplementary Information

Supplementary Information.

## Data Availability

The data that support the findings of this study are available from the corresponding author upon reasonable request.
