# Supplementary material for: Limited impact of bacterial virulence on early mortality risk factors in Acinetobacter baumannii bacteremia observed in a Galleria mellonella model
Source: Sci Rep. 2024 Jun 28;14:14960. doi: 10.1038/s41598-024-65940-2 (PMC11213897; doi:10.1038/s41598-024-65940-2)
Supplement: Supplementary file 1 — Supplementary Information. [file 41598_2024_65940_MOESM1_ESM.pdf]

# Supplemental Material

**Limited impact of bacterial virulence on early mortality risk factors in *Acinetobacter baumannii* bacteremia observed in a *Galleria mellonella* model.**

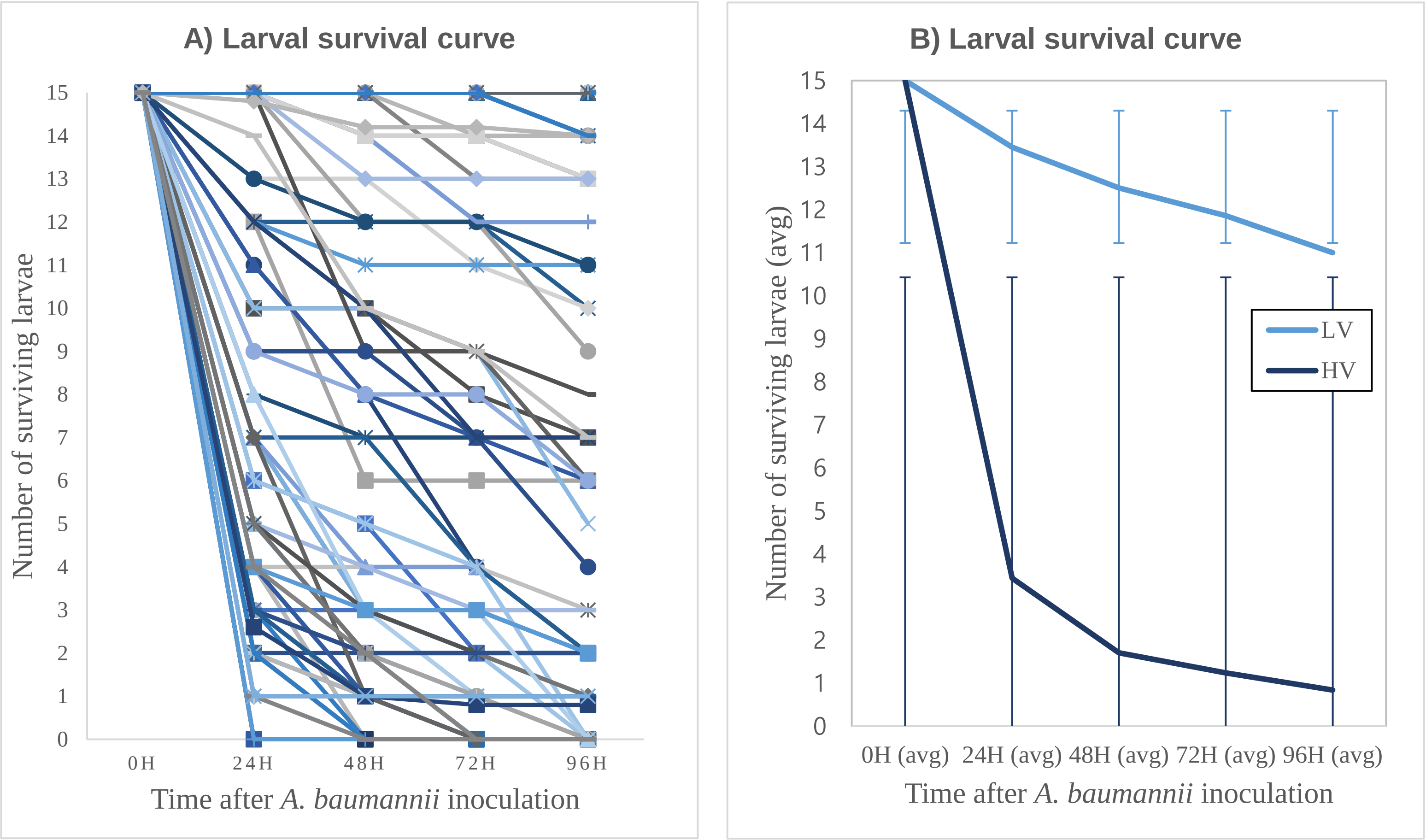


**Supplementary Figure S1.** Number of deaths of *Galleria mellonella* larvae according to the *Acinetobacter baumannii* inoculation time

A) Larval survival curves for all 92 collected strains. B) Mean larval survival curve according to the virulence groups. The black auxiliary line on the vertical axis indicates the standard deviation

avg, average; HV, high virulence; LV, low virulence

**Supplementary Table S1.** Comparison of the clinical characteristics of *Acinetobacter baumannii* bacteremia stratified by clinical isolates collection (n=138)

| | **Clinical isolates non-collected (n=46)** | | **Clinical isolates collected (n=92)** | | ***p*-value** |
|---|---|---|---|---|---|
| | **N** | **(%)** | **N** | **(%)** | |
| Age (y), mean±SD | 68.80±14.29 | | 68.53±13.52 | | 0.913 |
| Male sex | 30 | (65.2) | 61 | (66.3) | 0.899 |
| Acquisition site | | | | | 0.196 |
| Community acquired | 5 | (10.9) | 18 | (19.6) | |
| Nosocomial[a] | 41 | (89.1) | 74 | (80.4) | |
| CCI, mean±SD | 6.59±2.93 | | 6.23±2.85 | | 0.491 |
| Peptic ulcer disease | 3 | (6.5) | 4 | (4.3) | 0.583 |
| Myocardial infarction | 1 | (2.2) | 5 | (5.4) | 0.376 |
| Congestive heart failure | 4 | (8.7) | 5 | (5.4) | 0.465 |
| Peripheral vascular disease | 2 | (4.3) | 4 | (4.3) | 1.000 |
| Dementia | 3 | (6.5) | 6 | (6.5) | 1.000 |
| Chronic lung disease | 7 | (15.2) | 14 | (15.2) | 1.000 |
| Rheumatologic disease | 3 | (6.5) | 3 | (3.3) | 0.376 |
| Leukemia | 2 | (4.3) | 1 | (1.1) | 0.216 |
| Lymphoma | 1 | (2.2) | 2 | (2.2) | 1.000 |
| Solid organ cancer | 9 | (19.6) | 28 | (30.4) | 0.174 |
| Metastatic cancer | 10 | (21.7) | 11 | (12.0) | 0.131 |
| Chronic kidney disease | 13 | (28.3) | 24 | (26.1) | 0.786 |
| Cerebrovascular disease | | | | | 0.788 |
| No | 37 | (80.4) | 71 | (78.9) | |
| Yes | 4 | (8.7) | 11 | (12.2) | |
| Yes with hemiplegia | 5 | (10.9) | 8 | (8.9) | |
| Chronic liver disease | | | | | 0.192 |
| No | 41 | (89.1) | 72 | (78.3) | |
| CP-A | 0 | (0) | 4 | (4.3) | |
| CP-B, C | 5 | (10.9) | 16 | (17.4) | |
| Diabetes mellitus | | | | | 0.991 |
| No | 31 | (67.4) | 62 | (68.1) | |
| Yes | 7 | (15.2) | 14 | (15.4) | |
| Yes with complication | 8 | (17.4) | 15 | (16.5) | |
| HIV | 0 | (0) | 1 | (1.1) | 1.000 |
| Neutropenia[b] | 3 | (6.5) | 7 | (7.6) | 0.816 |
| Immunosuppressive agents | 16 | (34.8) | 21 | (22.8) | 0.135 |
| Recent chemotherapy | 8 | (17.4) | 9 | (9.8) | 0.200 |
| Systemic steroid | 9 | (19.6) | 12 | (13.0) | 0.315 |

| | | | | | |
|---|---|---|---|---|---|
| Other immunosuppressants | 2 | (4.3) | 2 | (2.2) | 0.598 |
| Transplantation | 2 | (4.3) | 1 | (1.1) | 0.216 |
| Pitt, mean±SD | 3.46±3.14 | | 4.6±3.62 | | 0.070 |
| Mechanical ventilation | 16 | (34.8) | 41 | (44.6 | 0.271 |
| Infection focus | | | | | 0.354 |
| Primary bacteremia | 10 | (21.7) | 15 | (16.3) | |
| Lung | 10 | (21.7) | 28 | (30.4) | |
| Central line-associated | 18 | (39.1) | 25 | (27.2) | |
| Pancreaticobiliary | 5 | (10.9) | 12 | (13.0) | |
| Intra-abdominal | 0 | (0) | 4 | (4.3) | |
| Urinary tract | 2 | (4.3) | 4 | (4.3) | |
| Others | 1 | (2.2) | 4 | (4.4) | |
| Mortality risk of infection focus | | | | | 0.320 |
| Low risk[c] | 20 | (43.5) | 32 | (34.8) | |
| High risk[d] | 26 | (56.5) | 60 | (65.2) | |
| Inppropriate empirical antibiotics | 27 | (58.7) | 61 | (66.3) | 0.381 |
| Infection focus removal | 7 | (15.2) | 17 | (18.5) | 0.634 |
| Carbapenem resistance | 22 | (47.8) | 57 | (62.0) | 0.114 |
| 7-day mortality | 14 | (30.4) | 34 | (37.0) | 0.681 |
| 30-day mortality | 18 | (39.1) | 45 | (48.9) | 0.552 |

SD, standard deviation; CCI, Charlson comorbidity index; CP, Child-Pugh class; SOFA, Sequential Organ Failure Assessment; CPR, cardiopulmonary resuscitation; BSI, bloodstream infection

[a]Nosocomial: 48 hours after hospital admission, 3 days after discharge, or 30 days after surgery.

[b]Neutropenia: Absolute neutrophil count <500/μL.

[c]Low risk infection focus: infection focus with associated mortality ≤30%, which included the urinary tract, intravenous catheter, and pancreaticobiliary tract.

[d]High risk infection focus: infection focus with associated mortality >30%, including the lungs, peritoneum, and unknown sources.

**Supplementary Table S2**. Antibiograms of *Acinetobacter baumannii* isolated from patients with *A. baumannii* bacteremia

| | Sensitive | | Resistant | | Intermediate | |
|---|---|---|---|---|---|---|
| | **N** | **%** | **N** | **%** | **N** | **%** |
| Amikacin (n=138) | 71 | 51.4 | 62 | 44.9 | 5 | 3.6 |
| Ceftazidime (n=137) | 54 | 39.4 | 77 | 56.2 | 6 | 4.4 |
| Cefepime (n=136) | 53 | 39.0 | 79 | 58.1 | 4 | 2.9 |
| Piperacillin/tazobactam (n=137) | 54 | 39.4 | 80 | 58.4 | 1 | 0.7 |
| TMP-SMX (n=132) | 63 | 47.7 | 69 | 52.3 | | |
| Ciprofloxacin (n=137) | 52 | 38.0 | 85 | 62.0 | | |
| Imipenem (n=138) | 60 | 43.5 | 78 | 56.5 | | |
| Meropenem (n=138) | 59 | 42.8 | 79 | 57.3 | | |
| Colistin (n=136) [a] | 135 | 99.3 | 1 | 0.7 | | |

TMP-SMX, trimethoprim-sulfamethoxazole

[a]An MIC of 2 µg/mL or less was determined to be susceptible using the criteria of the European Committee on Antimicrobial Susceptibility Testing (EUCAST)

**Supplementary Table S3.** Results of K-means clustering analysis according to the *Acinetobacter baumannii* virulence group classification

| | Number of surviving larvae, mean (SD) | |
|---|---|---|
| | High virulence n=50 | Low virulence n=42 |
| 0H | 15 (0.000) | 15 (0.000) |
| 24H | 3.43 (1.963) | 13.43 (2.307) |
| 48H | 1.70 (1.374) | 12.50 (2.907) |
| 72H | 1.24 (1.223) | 11.86 (3.475) |
| 96H | 0.84 (0.934) | 11.00 (4.061) |

SD, standard deviation

**Supplementary Table S4.** Comparison of Empiric Antibiotics Used in Patients with *Acinetobacter baumannii* Bacteremia: Early Mortality (n=48) vs. Non-early Mortality (n=90) Groups

| | Early mortality | | Non-early mortality | | *p*-value |
|---|---|---|---|---|---|
| | Number | (%) | Number | (%) | |
| No antibiotics used | 7 | (12.1) | 1 | (1.0) | 0.002 |
| 3rd cephalosporins | 4 | (6.9) | 35 | (34.0) | <0.001 |
| 4th cephalosporins | 0 | (0) | 2 | (1.9) | 0.543 |
| Imipenem | 0 | (0) | 1 | (1.0) | 1.000 |
| Meropenem | 18 | (31.0) | 7 | (6.8) | <0.001 |
| Ertapenem | 1 | (1.7) | 4 | (3.9) | 0.658 |
| Piperacillin/tazobactam | 8 | (13.8) | 20 | (19.4) | 0.439 |
| Fluoroquinolones[a] | 6 | (10.3) | 13 | (12.6) | 0.752 |
| Amikacin | 0 | (0) | 1 | (1.0) | 1.000 |
| Colistin | 6 | (10.3) | 9 | (8.7) | 0.653 |
| TMP-SMX | 3 | (5.2) | 0 | (0) | 0.040 |
| Other antibiotics | 5 | (8.6) | 10 | (9.7) | 0.900 |

TMP-SMX, trimethoprim-sulfamethoxazole

[a]The fluoroquinolone group includes ciprofloxacin and levofloxacin

**Supplementary Table S5.** Results of the univariate and multivariate analyses for risk factors of 30-day mortality in patients with *Acinetobacter baumannii* bacteremia (n=92)

| | **Univariate analysis** | | **Multivariate analysis** | |
|---|---|---|---|---|
| | **HR (95% CI)** | ***p*-value** | **adjusted HR (95% CI)** | ***p*-value** |
| Age | 1.025 (1.000–1.051) | 0.050 | 1.020 (0.993–1.48) | 0.143 |
| Sex | 0.680 (0.333–1.251) | 0.195 | | |
| CCI | 1.068 (0.975–1.170) | 0.159 | | |
| Immunosuppressed status[a] | 1.538 (0.805–2.935) | 0.192 | | |
| Pitt score | 1.363 (1.239–1.499) | **<0.001** | 1.336 (1.206–1.480) | **<0.001** |
| High-risk infection focus[b] | 2.152 (1.088–4.256) | **0.028** | 2.935 (1.253–6.874) | **0.013** |
| Inappropriate empirical antibiotics | 2.054 (1.039–4.063) | **0.038** | 1.629 (0.781–3.396) | 0.193 |
| Infection focus removal | 3.187 (1.139–8.918) | **0.027** | 1.771 (0.547–5.730) | 0.340 |
| Carbapenem resistance | 5.881 (2.478–13.956) | **<0.001** | 2.872 (1.147–7.196) | **0.024** |
| High virulence | 1.729 (0.983–3.187) | 0.080 | 1.075 (0.537–2.149) | 0.839 |

HR, hazard ratio; CI, confidence interval; CCI, Charlson comorbidity index; LV, low virulence; HV, high virulence

[a]Immunosuppressed status: absolute neutrophil count <500/µL, recent chemotherapy, use of steroids or immunomodulators.

[b]High-risk infection focus: infection focus with >30% associated mortality, including the lungs, peritoneum, and unknown sources.
